# Analysis on hydraulic characteristics of improved sandy soil with soft rock

**DOI:** 10.1371/journal.pone.0227957

**Published:** 2020-01-24

**Authors:** Yike Wang, Lei Ge, Shi Chendi, Huanyuan Wang, Jichang Han, Zhen Guo, Yangjie Lu

**Affiliations:** 1 Institute of Land Engineering and Technology, Shaanxi Provincial Land Engineering Construction Group Co., Ltd., Shaanxi, Xi’an, China; 2 Key Laboratory of Degraded and Unused Land Consolidation Engineering, the Ministry of Natural Resources of China, Shaanxi, Xi’an, China; 3 Shaanxi Provincial Land Consolidation Engineering Technology Research Center, Shaanxi, Xi’an, China; 4 Shaanxi Provincial Land Engineering Construction Group Co., Ltd., Xi'an, China; Central South University, CHINA

## Abstract

Hydraulic properties of sandy soil from the Mu Us sandy land of Shaanxi Province were analyzed by using SEM technology. Soil porosity, the water characteristic curve, and unsaturated hydraulic conductivity of aeolian sandy soil with added soft rock were analyzed, and fractal characteristics were established. Soil hydraulic properties revealed the effect of soft rock application on soil structure and hydraulic properties. Mass ratios of soft rock to aeolian sand were 1:5, 1:2, and 1:1. Results showed that the addition of soft rock can significantly increase the bulk density of sandy soil and reduce the total porosity and macroporosity. The mass fraction of water-stable aggregates greater than 0.25mm increases significantly, increasing the fractal dimension of soil pores; reducing the soil saturated water content and saturated hydraulic conductivity. SEM technology and pore fractal theory were used to predict the soil salinity curve and unsaturated hydraulic conductivity of the improved saline soil.

## Introduction

The various branches of earth science, including hydrogeology, soil science, environmental science, and geotechnical engineering, need to measure and estimate soil hydraulic properties (water characteristic curves, hydraulic conductivity, etc.)[1]. The methods for determining the soil hydraulic properties include direct measurement and indirect estimation. In actual operation, the soil hydraulic properties have large spatial variability, Direct measurements take a lot of time and require more complex testing skills, and the measured parameters often have problems such as low precision [2]. Since the 1970s, methods for indirectly estimating hydraulic properties based on soil properties have attracted widespread attention from scholars at home and abroad [3]. The use of fractal geometry methods to estimate soil hydraulic properties has been widely used in indirect methods. TYLER et al.[4] applied fractal dimensions for the first time to estimate the soil water characteristic curve. KRAVCHENKO et al.[5] proposed the fractal dimension calculation method for pore surface area using sponge fractal theory and improved the Brook-Corey model. The main difference between the fractal method and other indirect methods is that soil hydraulic characteristic parameters determined by the fractal method have clear physical meanings, and the fractal dimension can reflect the irregularity and complexity of the soil pores. At present, a large number of studies determine the fractal dimension of soil by particle size distribution, but the fractal dimension of soil is not only related to particle size distribution but also pore shape and arrangement[6–8]. However, the number, structure, and arrangement of soil pores are very complicated and hard to identify. The rapid development of SEM scanning and computer image processing technology can determine the structure of a sample without destroying the sample.

Mu Us Sandy Land is one of the four major sandy lands in China. The sandy land soil has high sand content, low clay content, and is loosely consolidated. With little or no structure, the sandy soil is highly permeable under both wet and dry conditions and retains little to no water. Existing remediation methods (including physical engineering methods, biological remediation methods, and chemical stabilization methods) could not resolve the desertification problem absolutely or change the structure of the sandy soil; however existing research shows that adding soft rock as a soil amendment can improve the physical characteristics of the sandy soil. Soft rock, also present in this area, the enrichment of silt and clay, not only gives the soft rock grains with fair retention capacity, but also can be used as amendment to improve the sandy soil. Soft rock mixed with sandy soil could break the compacted soft rock structure, as well as modify the loose sand in order to improve the water productivity and arability of the sand [9–11]. More than 1600ha of mixed soft rock and sandy soil exist in the Mu Us Sandy Land. The increased water retention capacity of the mixed soft rock and sandy soil could benefit plough layer soil and help control wind erosion[12].

Different ratios of soft rock to sandy soil have a significant influence on soil physical properties, water retention, and crop growth [13]. At present, there are few studies on the effects of different proportions of soft rock on the structure and hydraulic properties of aeolian sandy soil, and the research has not reached a consistent conclusion[14–16]. However, the structural and hydraulic properties of aeolian sandy soil can directly or indirectly affect other properties such as water and salt transport, nutrient retention and microbial activity in the soil. In order to promote soft rock as amendment to improved sandy soil, this paper takes the aeolian sandy soil in Mu Us Sandy Land of Shaanxi Province as the research object. Based on fractal theory and soil SEM technology, analysis of changes in soil properties such as soil porosity, water-stable aggregates, water characteristic curves, and unsaturated hydraulic conductivity of modified sandy soil, a fractal model was established to predict the soil hydraulic properties in the Mu Us Sandy Land of the Shaanxi Province to reveal the effects of soft rock on soil structure and hydraulic characteristics of aeolian sandy soil. Results of this study provide a theoretical basis for and new ideas for the estimation of soil hydraulic properties.

## Materials and methods

### Site

Soft rock and sand were collected from the Yuyang District, Yulin, China (108.58°E to 110.24°E, 37.49°N to 38.58°N). This research relies on the sand remediation demonstration project, and the project belongs to the Shaanxi Provincial Land Engineering Construction Group, the field studies did not involve endangered or protected species. The area is in a medium-temperate arid climate zone with the altitude ranges from 1000m to 1600m. 9 test plots were randomly placed on the Mu Us Sandy Land, meanwhile a 15m×12m sandy land was set as a control plot; The soft rock was first crushed until diameter less than 4, and then dumped in the test plot according to the quality of the aeolian sandy soil at 1:1, 1:2, 1:5. A construction machinery was used to plow the soil, so that the soft rock and sand were evenly mixed on the surface of the 30 cm tillage layer. The cultivation method of each plot is corn-potato rotation each year, all plots were repeated 3 times, and the planting is completed for one year. After that, the undisturbed soil was collected to determine the physical properties, and the undisturbed soil was collected to obtain an SEM image.

### Soil bulk density and porosity

Soil samples were collected using a 100cm^3^-ring cutter. Undisturbed soil samples were dried in a dry box at 105°C for 24 hours, and then the soil bulk density was measured. The total soil porosity was calculated using Eq 1.

$$P=1-\frac{\rho_{b}}{\rho_{B}}$$ (1)

where ρ_b_ is the soil dry bulk density, g·cm^-3^; ρ_B_ is the soil specific gravity, averaged 2.65g·cm^-3^; P is the total soil porosity.

### Water-stable aggregates

Water-stable aggregates of the soil were determined by wet sieving. Compound soil samples were air-dried and passed through 5mm and 2mm sieves, respectively. Soil samples were divided into three groups of 0-2mm, 2-5mm, >5mm, size ranges and then mixed with 20.0g of the undisturbed soil. Mixed soil samples were placed on a set of sieves having a combination of pore sizes of 5, 2, 1, 0.5, and 0.25mm. The sieves were slowly immersed in water, allowed to stand for 10 minutes, and then sieved at a rate of 30 times per minute for 5 minutes. After wet sieving, the aggregates on each of the sieves were separately washed into an aluminum box and dried for weighing, and the mass fraction of each of the soil samples was calculated.

### Soil hydraulic parameters

The saturated hydraulic conductivity of the soil was determined using the fixed head method. The soil moisture characteristic curve was determined using the suction plate method, with suction values of 0, 15, 30, 60, 100, 300, 600, 900cm (see S1 Table). The obtained soil moisture characteristic curve was input into RETC, A nonlinear least-squares optimization program which uses empirical relationships for describing the water-retention curve and predictive models for characterizing the saturated hydraulic conductivity distributions and fitted using the classical single-peak van Genuchten model to obtain the hydraulic parameters of the test soil.

### SEM images acquisition

Due to the high sand content in the samples, the injection method was used to cure the sample, and the cemented samples were polished to 30 μm thick sheets. The FEI environmental scanning electron microscope(ESEM) Q45 (magnification 100,000 times, resolution 3.0 nm· kv^-1^) was used to obtain the SEM images of the four soil samples. In order to make the analysis comparable, the selected images have a magnification of 1 000 times, the image resolution is 0.095 μm pixel^-1^, and the analysis area is 127.8 μm × 95.8 μm.

### SEM images analysis

**Fig 1:**
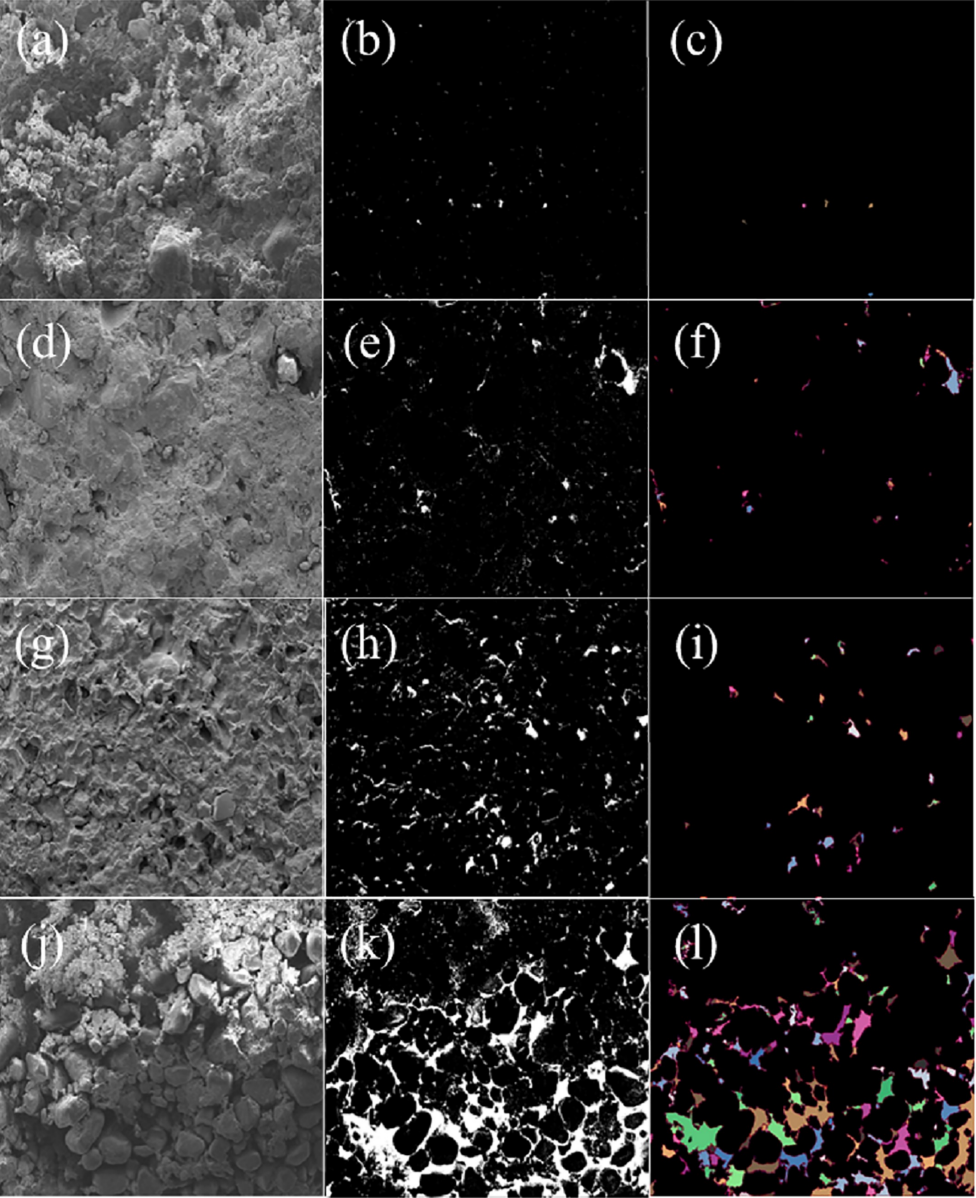
The SEM images of soft rock and sand compound soil with different mixing ratio. Fig 1A, 1D, 1G and 1J are the SEM images of soft rock and sand compound soil 0:1, 1:5, 1:2, 1:1,respectively; Fig 1B, 1E, 1H and 1K are the pore distribution of soft rock and sand compound soil 0:1, 1:5, 1:2, 1:1,respectively; Fig 1C, 1F, 1I and 1L are the macropore distribution of soft rock and sand compound soil 0:1, 1:5, 1:2, 1:1, respectively.

The obvious depressions or protrusions in the original image were removed, and the exposure, brightness, contrast and gamma correction values were adjusted to make the image reach the best analysis state by using the Photoshop software. The Pores and Cracks Analysis System (PCAS) can automatically quantitatively analyze statistical parameters such as number, area, length, width, orientation, shape coefficient, and area probability distribution index in images, as well as display various geometric parameters of all particles and pores in the data sheet(see **Fig 1**).

### Determination of the fractal dimension

The Sandbox method was used to measure the fractal dimension [17]: a series of increasing boxes or circles were used to cover the pores, and the numbers of target pixels in different measurement ranges were counted. The boxes with lengths of 50, 100, 150, 200, 300, and 400 pixels were selected for counting, and the centroid of the boxes were selected at the center point of the image (265, 265). The maximum size of the method is limited by the maximum size of the image. The scale invariance of ε and N is considered, as follows:

N(ε)∝εD (2)

Where ε is the measurement unit size; N is the number of pixels in different boxes; D is the fractal dimension.

### Fractal model of soil water characteristic curve

For soils with a fractal pore size distribution, AVNIR stablished a theoretical prediction model between pore volume and radius, and results show that pore volume is[18]

$$-\frac{dV(>r)}{dr}\propto r^{E-D}$$ (3)

In the formula (3):

where r is the pore radius; V is the pore volume with radius greater than r; D is the Fractal dimension describing the pore size distribution; E is the topological dimension in Euclidean geometry, equal to 2 or 3.

The integral form of Eq (3) is:

$$V(>r)=-\beta r^{E-D}+V_{0}$$ (4)

Where β is a constant, and Eq (4) is the basic formula for studying the hydraulic properties of soil using fractal theory.

According to the capillary rise equation (Young-Laplace equation), the expression of the pore radius r and the corresponding water suction φ is(5)

$$\varphi (r)=\frac{2\gamma \;\cos\theta }{\rho_{w}gr}$$ (5)

where φ(r) is the capillary pressure with a pore radius of r; γ is the surface tension coefficient of water; φ is the contact angle of water with the surface of the pores; ρ_w_is the density of water; g is the acceleration due to gravity.

After the pressure φ(r) is applied, all pore waters at a radius greater than r are discharged, and all pores within a radius less than r are filled with water.

According to Menger sponge fractal theory, KRAVCHENKO[5] derived the relationship between the fractal dimension Ds of the pore surface and the particle radius and mass fraction, and brought the Ds into the exponential term of the Brook-Corey model to solve the soil water characteristic curve.

The KZ-BC model is given by:

$$\theta (h)=\theta_{r}+(\theta_{s}-\theta_{r}){\left(\frac{h_{a}}{h}\right)}^{3-D_{3-D}}$$ (6)

where h is the soil suction, cm; θ(h) is the soil moisture content, cm^3^·cm^-3^; θ_s_ is the soil saturated water content, cm^3^·cm^-3^; θ_r_ is the soil residual moisture content, cm^3^·cm^-3^; h is the intake value, cm,Αh and the shape parameter α are reciprocal to each other; D_3-D_ is the Fractal dimension of pore surface in three-dimensional space.

In this paper, the soil moisture characteristic curve is simulated using formula (6).

### Classic model of hydraulic conductivity

Unsaturated hydraulic conductivity is difficult to directly measure, so it is estimated primarily using soil water characteristic curves and saturated hydraulic conductivity. The van Genuchten model[19] formula for the soil water characteristic curve is

$$\frac{\theta (h)-\theta_{r}}{\theta_{s}-\theta_{r}}={\left[\frac{1}{1+{\left(\alpha h\right)}^{n}}\right]}^{m}$$ (7)

where α is the the reciprocal of the intake value, cm^-1^; n, m is the shape parameter, m = 1–1 / n;

The soil nutrient curve model and Mualem derived by van Genuchten combined with the water conductivity model give the formula for calculating the unsaturated hydraulic conductivity K(θ)

$$\frac{K(\theta )}{K_{s}}=\sigma^{1/2}{\left[1-{\left(1-\sigma^{1/m}\right)}^{m}\right]}^{2}$$ (8)

$$\sigma =\frac{\theta -\theta_{r}}{\theta_{s}-\theta_{r}}$$ (9)

where (8):K(θ) is the unsaturated hydraulic conductivity, cm·h^-1^; Ks——saturated hydraulic conductivity, cm·h^-1^; σ is the dimensionless moisture content.

### Fractal model of hydraulic conductivity

The widely used hydraulic conductivity model is the Mualem model:

$$\frac{K(\theta )}{K_{s}}=\theta^{1/2}\left[\int_{0}^{\theta \prime }\frac{1}{h^{2}(x)}dx/\int_{0}^{1}\frac{1}{h^{2}(x)}dx\right]$$ (10)

$$\theta^{\prime }=\frac{\theta }{\theta_{s}}$$ (11)

The Sierpinski carpet model was used to obtain a fractal model of soil water characteristic curve[20].

$$\frac{\theta }{\theta_{s}}={\left(\frac{h}{h_{a}}\right)}^{D_{2-D}-2}$$ (12)

Substituting Eq (12) into the Mualem model gives the fractal form of the Mualem model.

$$\frac{K(\theta )}{K_{s}}={\left(\frac{\theta }{\theta_{s}}\right)}^{\frac{\frac{5D_{2-D}}{2}-7}{D_{2-D}^{-2}}}={\left(\frac{h}{h_{a}}\right)}^{\frac{5D_{2-D}}{2}-7}$$ (13)

where D_2−D_ is the two-dimensional space pore fractal dimension.

According to the cross-section agreement, the relationship between the three-dimensional space fractal dimension D_3-D_ and the two-dimensional space fractal dimension D_2−D_ is D_2−D_ = D_3-D_-1. This paper uses formula (13) to analyze and predict soil hydraulic conductivity. Model prediction results were evaluated using the root mean square error (RMSE).

## Results

### Effect of applying soft rock on aeolian sandy soil structure and hydraulic characteristics

The application of different mass ratios of soft rock significantly affected soil bulk density (P<0.05)(see **Table 1**). The soil bulk density of the control group was 1.30 g·cm^-3^, which was significantly lower than the soil bulk density after soft rock application. Correspondingly, the application of soft rock with different mass ratios (1:1, 1:2, and 1:5) also had a significant effect on soil total porosity (P < 0.05).The total porosity of the soil applied by different mass ratios soft rock(1:1, 1:2, and 1:5) was 45.66%, 43.40% and 42.23%, which was significantly lower than 50.94% of the control group. At the same time, the application of soft rock increased significantly in the soil by more than 0.25 mm. The mass fraction of water-stable aggregates (P<0.05), the mass fraction of water-stable aggregates in the control group was greater than 0.25 mm, and the mass fraction of water-stable aggregates was 15.50%, which increased by 18.50%, 39.03%and 82.06% after application of different mass ratios (1:1, 1:2, and 1:5) soft rock. In addition, the correlation coefficient between soil total porosity and large aggregates reached 0.91.

**Table 1:** Structural parameters of applying different amounts of soft rock.

M(Feldspathic sandstone):m(Aeolian sandy soil)	Bulk weight / (g·cm^-3^)	Total porosity/%	> 0.25mm water stable aggregates mass fraction /%	Macrospores porosity/%	D_3-D_
0:1	1.30b	50.94b	15.50b	15.35	2.623
1:5	1.44a	45.66a	18.38a	7.54	2.729
1:2	1.50a	43.40a	21.55a	0.67	2.779
1:1	1.56a	41.13a	28.22a	0.13	2.812
Correlation	-100%	-	-91.7%	86.1%	-94.8%

The application of soft rock increased soil pore fractal dimension and decreased macrospores porosity (P < 0.05). The macrospores porosity of the control group was 15.35%, and the addition of soft rock (1:5, 1:2 and 1:1) decreased the soil macrospores porosity by 50.87%, 95.65% and 99.15%. The fractal dimension of soil applied with different mass ratios (1:1, 1:2, and 1:5) soft rock were 2.729, 2.779, and 2.812, which were significantly higher than the fractal dimension of the control group 2.623. The correlation coefficient between soil total porosity and large porosity reached 0.88.

The soil moisture characteristic curve fit by the van Genuchten model has a correlation coefficient greater than 0.90 (see **Table 2**). The shape parameters α, m, and n of the model are slightly reduced, and the soil saturated water content is significantly different (P < 0.05) after soft rock application. After adding soft rock with different mass ratios (1:1, 1:2, and 1:5), the soil saturated water content was 0.3834 cm^3^·cm^-3^, 0.4187 cm^3^·cm^-3^, and 0.4187 cm^3^·cm^-3^ respectively, which was significantly lower than the saturated water content of the control group (0.4287 cm^3^·cm^-3^). With increasing amounts of soft rock, the saline soil saturated hydraulic conductivity decreased. The saturated hydraulic conductivity of the control group was 736.9cm·d^-1^. For mass ratios of soft rock to aeolian sandy of 1:1, 1:2, and 1:5, the respective soil saturated hydraulic conductivity decreased to 77.8%, 93.39%, and 92.57%.

**Table 2:** Hydraulic parameter of applying different amounts of soft rock.

Soft rock: Aeolian sand	$\frac{\theta -\theta_{r}}{\theta_{s}-\theta_{r}}={\left(\frac{1}{1+{\left(\alpha h\right)}^{n}}\right)}^{m}$					K_s_ /(cm·d^-1^)	R^2^
	*θ*_*r*_ /(cm^3^·cm^-3^)	*θ*_*s*_ /(cm^3^·cm^-3^)	α	n	m		
0:1	0	0.4287	0.1403	2.1076	0.5255	736.9	0.9757
1:5	0	0.3834	0.1251	1.5457	0.3530	163.13	0.9362
1:2	0	0.4187	0.1543	1.4980	0.3324	48.69	0.9245
1:1	0	0.3728	0.0926	1.5598	0.3589	54.69	0.9102

### Soil moisture characteristic curve model verification

**Fig 2:**
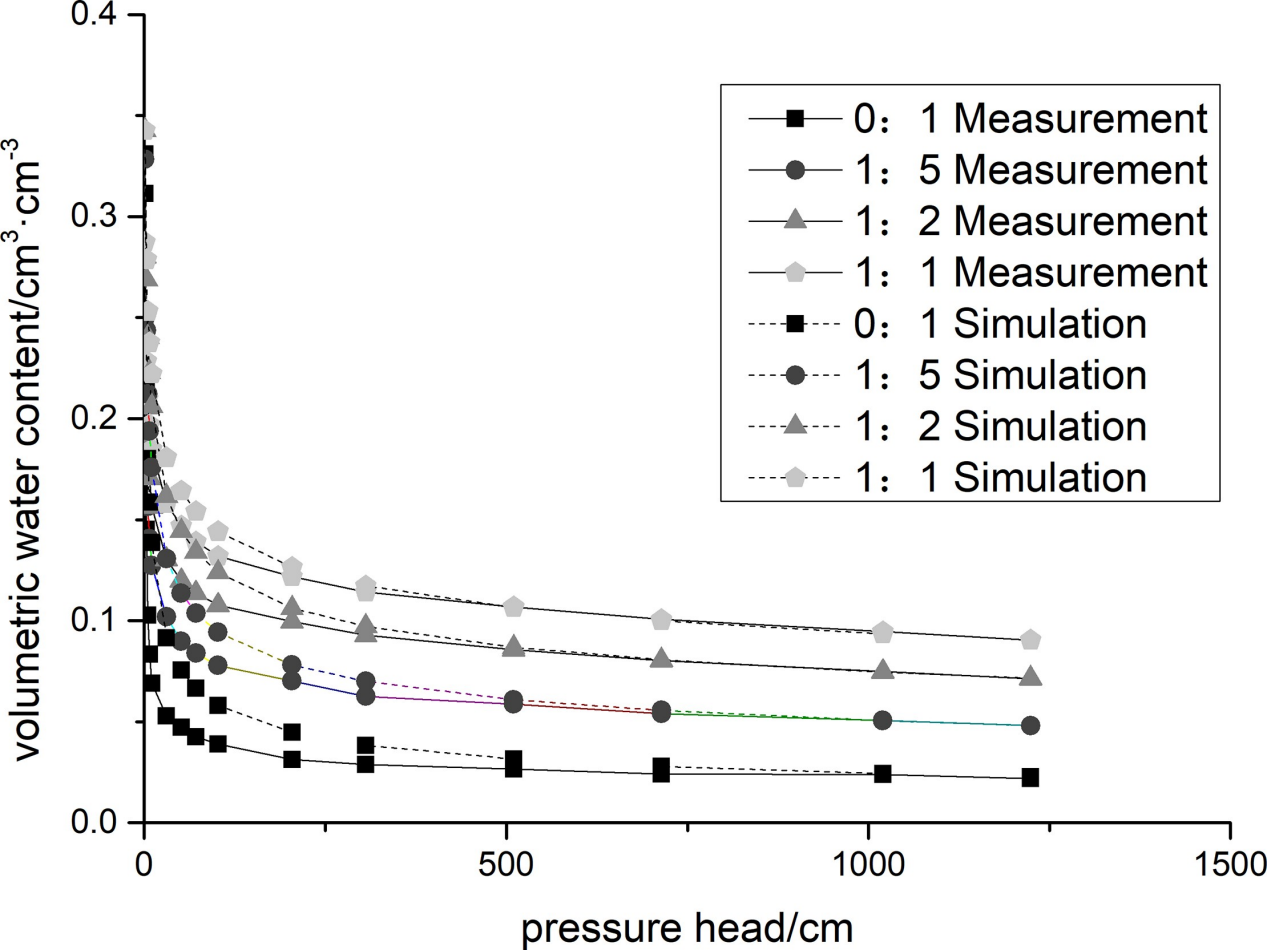
Comparison of predicted and measured soil water characteristic curves after soft rock application.

The soil moisture characteristic curve was simulated using formula (6), the KZ-BC model. Parameters values in the formula are given in **Table 1** and **Table 2**. Fractal model prediction values for the improved aeolian soil moisture characteristic curves for treatments with different soft rock mass ratios were calculated. Measured values are shown in **Fig 2**.

Results show that soil water characteristic curves under different soft rock treatments are consistent. Under low pressure, the volumetric water content decreases increasing suction. However, the addition of soft rock significantly increased the soil water holding capacity. Under the same suction, the soil water holding capacity of the soil with added soft rock was significantly higher than the control group.

Predicted results of the KZ-BC model are consistent with the measured values, but different mass ratios soft rock (1:5, 1:2, 1:1) were added to the KZ-BC model at low suction in the case of lower soil volumetric water content estimates. The KZ-BC fractal model was used to simulate the soil moisture characteristic curve R^2^ (see **Table 3**), and the model can meet the accuracy requirements. The RMSE between the water characteristic curve predicted by the fractal model and the measured curve is shown in **Table 3**. The smaller the RMSE value is, the more accurate the model prediction will be.

**Table 3:** Precision parameters of fractal model predicting soil properties.

Feldspathic Sandstone: Aeolian sand	Soil moisture characteristic curve		Unsaturated hydraulic conductivity	
	R^2^	RMSE /(cm^3^·cm^-^^3^)	R^2^	RMSE /(cm^3^•cm^-^^3^)
0:1	0.897	0.0024	0.973	0.09
1:5	0.994	0.0043	0.998	0.15
1:2	0.987	0.0067	0.979	0.52
1:1	0.997	0.0034	0.987	0.15

### Soil hydraulic conductivity model verification

**Fig 3:**
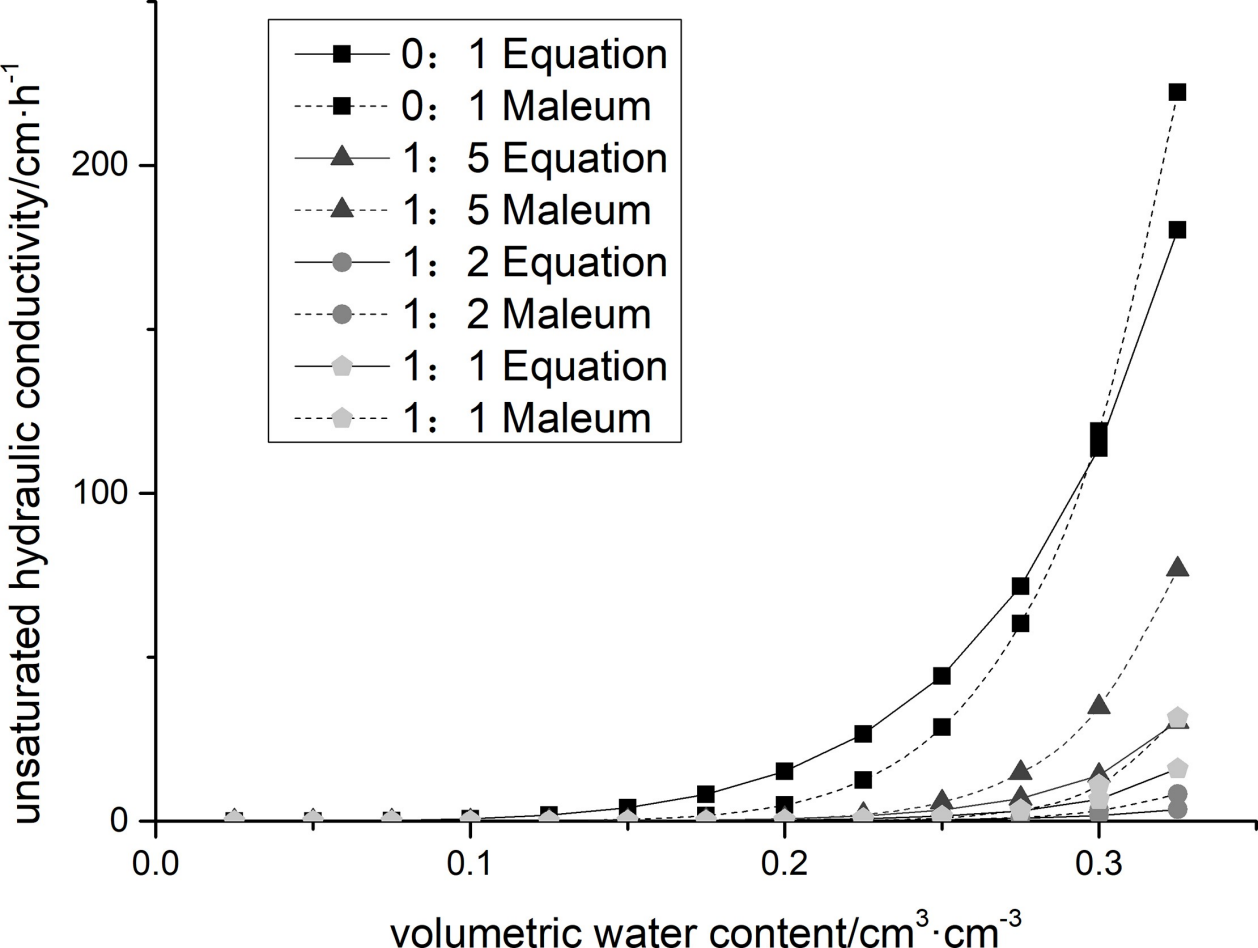
Comparison of predicted and measured parsing hydraulic conductivity after soft rock application.

Based on the measured soil water characteristic curves and saturated hydraulic conductivity, soil hydraulic conductivity was calculated using Eq (8), and soil hydraulic conductivity was predicted using the Mualem fractal model (13) (see **Table 1** and **Table 2**). The unsaturated soil hydraulic conductivity analytical values and predicted values of 1:1, 1:2, and 1:5 for soft rock modified with sandy soil are shown in **Fig 3**.

The change trend of unsaturated hydraulic conductivity for different soft rock treatments is the same, that is, as the volumetric water content increases, the unsaturated hydraulic conductivity increases rapidly (see **Fig 3**). The addition of soft rock increases soil water conductivity, especially in near-saturated soil. The Mualem fractal model was used to predict the soil unsaturated hydraulic conductivity R^2^ (see **Table 3**), and the model accuracy meets the requirements. The unsaturated hydraulic conductivity curve was obtained from analysis of soil moisture characteristic curve and saturated hydraulic conductivity. The RMSE between the unsaturated hydraulic conductivity curves predicted by the Mualem fractal model is shown in **Table 3**.

## Discussion

### Effect of applying soft rock on soil structure and hydraulic characteristics of aeolian sandy soil

Results of this test show that the structure of aeolian sandy soil can be achieved 1 year after soft rock application (see **Table 1**). Soft rock application can significantly increase the bulk density of saline soil. Relatively speaking, the soft rock has a large bulk density and a dense texture. The test sand has a small bulk density. Adding soft rock to the sand can improve the soil tightness and increase soil bulk density. Moreover, the application of soft rock promotes soil particle agglomeration, which is beneficial for the formation of multi-level pores between the aggregates.

The addition of soft rock significantly increases the soil saturated water content. This may be because the addition of soft rock significantly increases the number of large aggregates, so that the soil specific surface area increases and water retention capacity increases[21]. Similarly, the addition of soft rock will reduce the soil saturated hydraulic conductivity, possibly due to the more abundant pore structure after the soft rock is added. The poor air permeability and water permeability of the soft rock itself can improve water retention in the sandy soil, and reduce saturated water conductivity. The application of soft rock can increase the moisture content of sand[22]. The treatment of biomass carbon to soil volume ratio of 25.0% can significantly increase the soil moisture content in the root zone of the grassland, while the soil saturated water conductivity decreased with increasing biomass carbon application[23]. The observed influence of soft rock on soil hydraulic properties is consistent with the conclusions of Omoro et al[24]. Experimental results showed that the application of soft rock can effectively improve soil structure and hydraulic properties. Compared with organic soil improver, soft rock application has lower cost and can be applied to a large area. Compared with the grass-grid method for improving aeolian soil, soft rock application can effectively improve the structure and hydraulic characteristics of saline soil. Therefore, it is of great significance to study the application of soft rock to improve aeolian sandy soil hydraulic properties. This study only examined the changes in soil structure and hydraulic characteristics of aeolian sandy soil after adding soft rock for 1 year. A long-term positioning experiment was proposed to explore the influence of soft rock on the characteristics of aeolian sandy soil for longer time periods.

### Prediction of hydraulic properties of aeolian sandy soil based on SEM technology and fractal theory

Experimental results showed that the combination of SEM technology and fractal theory model can accurately predict the hydraulic properties of soft rock improved aeolian sandy soil. However, the fractal dimension of soil is not only related to particle size, but also to particle shape and arrangement. Therefore, the fractal dimension of soil is estimated using particle size. Obviously this method is not complete. The image processing software used in this experiment analyzes the soil pore fractal dimension, which can reflect the soil pore state more completely than the previous fractal dimension estimated using particle size. The fractal dimension obtained in this experiment predicts the hydraulic properties of soil with high precision. Compared with traditional fractal dimension prediction particle size estimation methods, this new method is fast and accurate. For soil water characteristic curve prediction, the addition of different mass ratios soft rock (1:5, 1:2, and 1:2) makes the prediction result of the KZ-BC model as a whole larger than the measured value, which may be due to the soil after soft rock addition. The pore structure changes significantly, and under different pressures, the pores affecting the soil moisture content are different. In KZ-BC model, the complete pressure head section uses a fractal dimension to describe the effect of pores on soil moisture, thus making the fractal dimension Ds too large. Under the same pressure head, the soil moisture content of the KZ-BC model is higher than the actual soil moisture content. Later studies should consider classifying pores and calculating their fractal dimensions to predict soil hydraulic properties.

When predicting unsaturated hydraulic conductivity, the fractal model overestimates the soil unsaturated hydraulic conductivity. This may be because the entire soil layer is not completely uniform, and there are some dense layers that complicate water flow. In this case, one fractal model may not be sufficient. Although the scanning technology and the fractal model are used to simulate the hydraulic properties of the soil and good simulation results are obtained, the model can be further optimized in the future, and the soil properties can be fully considered to make the model prediction more accurate.

## Conclusion

Applying different mass ratios of soft rock (1:5, 1:2, and 1:1) significantly increased the soil bulk density of aeolian sandy soil, reduced soil total porosity and macro porosity, promoted the formation of water-stable aggregates, and increased soil bulk density, Pore fractal dimension, reduced soil saturated water content and saturated hydraulic conductivity. Combining SEM technology and pore fractal theory to predict the soil moisture characteristic curve and unsaturated water conductivity of improved aeolian sandy soil is accurate and faster than the traditional particle size estimation fractal dimension method.

## Supporting information

S1 Table The water characteristic curve of soft rock and sand compound soil with different mixing ratios. (XLS)
